# Loop-Mediated Isothermal Amplification for Detection of Plant Pathogens in Wheat (*Triticum aestivum*)

**DOI:** 10.3389/fpls.2022.857673

**Published:** 2022-03-15

**Authors:** Sandra V. Gomez-Gutierrez, Stephen B. Goodwin

**Affiliations:** ^1^Department of Botany and Plant Pathology, Purdue University, West Lafayette, IN, United States; ^2^USDA-Agricultural Research Service, West Lafayette, IN, United States

**Keywords:** loop-mediated isothermal amplification, wheat, diseases, diagnosis, detection, quantification

## Abstract

Wheat plants can be infected by a variety of pathogen species, with some of them causing similar symptoms. For example, *Zymoseptoria tritici* and *Parastagonospora nodorum* often occur together and form the Septoria leaf blotch complex. Accurate detection of wheat pathogens is essential in applying the most appropriate disease management strategy. Loop-mediated isothermal amplification (LAMP) is a recent molecular technique that was rapidly adopted for detection of plant pathogens and can be implemented easily for detection in field conditions. The specificity, sensitivity, and facility to conduct the reaction at a constant temperature are the main advantages of LAMP over immunological and alternative nucleic acid-based methods. In plant pathogen detection studies, LAMP was able to differentiate related fungal species and non-target strains of virulent species with lower detection limits than those obtained with PCR. In this review, we explain the amplification process and elements of the LAMP reaction, and the variety of techniques for visualization of the amplified products, along with their advantages and disadvantages compared with alternative isothermal approaches. Then, a compilation of analyses that show the application of LAMP for detection of fungal pathogens and viruses in wheat is presented. We also describe the modifications included in real-time and multiplex LAMP that reduce common errors from post-amplification detection in traditional LAMP assays and allow discrimination of targets in multi-sample analyses. Finally, we discuss the utility of LAMP for detection of pathogens in wheat, its limitations, and current challenges of this technique. We provide prospects for application of real-time LAMP and multiplex LAMP in the field, using portable devices that measure fluorescence and turbidity, or facilitate colorimetric detection. New technologies for detection of plant pathogen are discussed that can be integrated with LAMP to obtain elevated analytical sensitivity of detection.

## Introduction

Wheat is one of the world’s most important cereal crops, with global production estimated at 762.6 million tons for 2020 and a growing area of 222.28 million hectares (Manjunatha et al., 2018). Wheat plants can be infected by a variety of fungal pathogens, which cause losses totaling about 20% of global production (Kuzdraliński et al., 2017). The top 10 fungal diseases of wheat include rusts (caused by *Puccinia* spp.), the Septoria leaf blotch complex (*Zymoseptoria tritici* and *Parastagonospora nodorum*), powdery mildew (*Blumeria graminis*), wheat blast (*Pyricularia oryzae* Triticum lineage), and several afflictions incited by species of Fusarium (Serfling et al., 2017; Thierry et al., 2020). Regionally important fungal pathogens include *Pyrenophora tritici-repentis*, which causes tan spot, and *Bipolaris sorokiniana* (formerly known as *Cochliobolus sativus*), the causal agent of spot blotch disease (Serfling et al., 2017). The species of rust pathogens that affect wheat include *Puccinia striiformis* f. sp. *tritici*, which causes stripe rust, *P. graminis* f. sp. *tritici*, the cause of stem rust, and *P. triticina* (synonym: *P. recondita* f. sp. *tritici*), which causes leaf rust and is the most widely distributed (Figueroa et al., 2018).

The Septoria leaf blotch pathogens, *Zymoseptoria tritici* (cause of Septoria tritici blotch) and *Parastagonospora nodorum* (incitant of Stagonospora nodorum leaf and glume blotch), form a major disease complex of wheat that affects worldwide production and causes up to 25% yield losses in numerous countries, such as Germany (Tian et al., 2005). In the United Kingdom, Septoria tritici blotch (STB) causes 10%–20% average annual losses (Fones and Gurr, 2015). This disease can reduce yield in durum wheat by 40% in Tunisia (Bel Hadj Chedli et al., 2020), while in Morocco, Algeria, and other Mediterranean regions severe epidemics reduce yield in bread and durum wheat by 35%–50% (Siah et al., 2014; Bel Hadj Chedli et al., 2020). In the worst-affected areas of Ethiopia, STB causes 25%–82% losses (Mekonnen et al., 2020). Septoria nodorum blotch (SNB) causes up to 31% yield losses in European regions (Downie et al., 2018) and around 9% yield loss across the wheat belt of Western Australia (Shankar et al., 2021).

Wheat blast caused by *Pyricularia* (formerly *Magnaporthe*) *oryzae* Triticum lineage is also a major threat that can cause total crop losses. This disease has not been reported yet in the United States. However, the widespread dissemination of this pathogen to major wheat-producing areas of the world caused by global trade is a major concern because of the seedborne nature of the *P. oryzae* Triticum lineage (Goulart and Paiva, 1990).

Currently, detection of pathogens and assessment of resistance in wheat plants depend mostly on visual or microscopic examination of the symptoms. Because similar symptoms can be caused by *Z. tritici*, *P. nodorum*, and other leaf pathogens, such as *P. oryzae* or *Pyrenophora tritici-repentis*, accurate detection of these pathogens can be challenging, particularly when they occur together. Moreover, some symptoms are often non-specific and may be confused with lesions associated with biotic stress or normal development, such as natural senescence, with which the coalescing lesions caused by *P. nodorum* can be easily confused (Tian et al., 2005).

Fast and accurate detection of wheat pathogens is required to limit and prevent their spread into disease-free regions (Cruz and Valent, 2017). Accurate detection of pathogens in wheat is a crucial step in applying the most appropriate disease management strategy based on the biology of the causal agents. Morphological and microscopic approaches to identify plant pathogens in wheat require taxonomic knowledge and time to determine the causal agent of observed symptoms. Although these methodologies are an important part of disease diagnostics, they can give unreliable results due to the large number of pathogens and symptoms, and the need for trained experts (Aslam et al., 2017). In recent years, a variety of molecular tools has been developed for fungal detection and identification. These include immunological methods, also known as serological assays, and nucleic acid-based techniques, which are predominantly based on the PCR (Lazcka et al., 2007; Aslam et al., 2017).

PCR-based methods were the foundation to develop numerous DNA-based approaches for plant pathogen detection that give reliable results. Multiple modifications and improvements to PCR have been developed that increase efficiency of the technique. The development of real-time quantitative PCR allowed both detection and quantification of the pathogens, which is relevant to determine the severity of infections (Lau and Botella, 2017). However, PCR-based methods involve challenges for multiple plant pathogen detection and field applications. The equipment required along with reaction time and high-temperature requirements demand the application of other techniques that overcome these difficulties and are suitable for field application.

Many isothermal (constant temperature) amplification techniques have been developed that can be more easily applied in the field. One of the most promising of these is loop-mediated isothermal amplification (LAMP). This approach was first developed by Notomi et al. (2000) and was rapidly adopted for detection of plant pathogens due to its high specificity, sensitivity, efficiency, and isothermal conditions that can be conducted in the field (Iwamoto et al., 2003). In this review, we explain the LAMP principles and LAMP-based approaches. Then we focus on the application of LAMP for detection of fungal pathogens and viruses in wheat, including a compilation of studies that demonstrate its application. We discuss the advantages and disadvantages of LAMP for field applications, compare LAMP against other isothermal-based techniques and present different alternatives for LAMP to be applied as a field assay.

## Conventional Methods for Plant Pathogen Detection

### Morphological Methods

Microscopic evaluation of characteristic morphological features of plant pathogens is a traditional detection method. This approach relies on initial pathogen isolation on selective culture media that supplies the nutritional requirements of the microorganism. Obligate pathogens must be grown on their host species, which can be inconvenient and time-consuming (Buja et al., 2021). This is followed by observation of colony appearance and morphological attributes of structures, such as spores, mycelia, and fruiting bodies in fungal organisms through microscopy techniques (Mahlein, 2016). The interpretation of visual symptoms in the host is a tool to verify the causal agent, although multiple plant pathogens can co-exist and cause disease to the plants, which makes accurate detection a difficult task. Disadvantages of these approaches include the laboriousness of pathogen isolation and growth on culture media and the production of structures. For instance, colonies must experience specific conditions to produce spores, which may cause delays in laboratory work flow, and some fungal reproductive structures are not always produced on culture media (Buja et al., 2021). Nowadays, detailed guidelines and standards are available for visual identification. However, recognition of morphology in pathogen structures relies on the human eye and the training and taxonomical knowledge of the field expert, which can lead to misleading conclusions.

### Microbiological Methods

Microbiological approaches require culturing of the pathogen on various appropriate artificial and selective media under a variety of conditions, which is followed by microscopic techniques (Ward et al., 2004). Microscopic observation is accompanied by examination of structures by staining. For instance, the gram stain is one of the most useful tools to differentiate bacteria beyond the genus level. Biochemical and selective tests based on degradation of particular substrates and nutritional requirements can be applied to differentiate particular species (Lazcka et al., 2007). Production and characteristics of sporulating structures in fungi, as well as biochemical-based methods, are used for pathogen detection to yield better results (Ray et al., 2017). Conventional microbiology methods are still the simplest and least expensive. However, culturing of pathogenic species often requires days or weeks to complete. This is a major disadvantage when accurate and rapid diagnosis is required. Also, the results might not always be conclusive, as traditional methods are not highly sensitive and they are not always suitable for detection of pre-symptomatic infections (Ward et al., 2004).

### Serological Assays

The ELISA approach relies on interaction between antigens from pathogens and specific antibodies. This technique is fast and simple to implement and interpret its results, which makes it frequently used for high-throughput testing. Use of monoclonal and recombinant antibodies is an improvement of ELISA applications because it increases the sensitivity and specificity of the assays (Boonham et al., 2014). ELISA is the most widely used and cost-effective serological technique for diagnostics due to its high sensitivity (Khater et al., 2017). However, important limitations of ELISA are low availability of enzyme-conjugated antibodies and low specificity (Tomlinson and Boonham, 2008; Khater et al., 2017). Additionally, the production of monoclonal antibodies may be expensive (Baldi and La Porta, 2020).

ELISA requires high-quality antisera to achieve a sensitive and specific binding to viral antigens. Generation of such antisera requires purification of virions and other viral proteins as antigen. This requires high virology expertise (Boonham et al., 2014). Nevertheless, double-antibody sandwich (DAS)-ELISA, which involves enzyme attachment to the antibody probe has been reported for pathogen detection. Moreover, in the indirect method termed DASI-ELISA, the antibody probe remains unlabeled and, instead, the enzyme is attached to a second antibody specific to the probe antibody. DASI-ELISA has greater sensitivity and convenience compared to DAS-ELISA (Rowhani et al., 2005). Other modifications, such as triple-antibody sandwich (TAS)-ELISA and Dot-ELISA (where reagents are spotted onto a surface such as a nitrocellulose membrane), increased the efficiency of the technique and provide alternatives for detecting plant viruses. Antibodies against viral pathogens in plants are available and the techniques can be adapted for diagnosis of plant viruses that affect wheat (Gao and Wu, 2022).

### PCR Assays

DNA-based identification tools have provided researchers and farmers with plant pathogen detection techniques that are both precise and reliable (Aslam et al., 2017; Thierry et al., 2020). PCR is the most common DNA amplification technique for detection of plant pathogens (Lau and Botella, 2017). It is 100 times more sensitive than serological methods and can provide both qualitative and quantitative results, the latter when coupled with ability to detect DNA in real time (Ray et al., 2017). Real-time PCR follows the same procedures as the conventional approach but includes quantification in real time of amplified DNA products, using a fluorescent marker that binds to the DNA (Alemu, 2014). All PCR techniques employ high heat to obtain a single-stranded target DNA by denaturation of the double-stranded template, which requires expensive equipment (Wong et al., 2018). This is an obvious limitation of using PCR for detection of plant pathogens in the field (Khater et al., 2017).

### Isothermal Amplification Techniques

In contrast to PCR, which requires cycling between a high temperature for DNA denaturation and lower temperatures for primer annealing and DNA synthesis, many techniques are available in which DNA amplification can occur at a single, constant (isothermal) temperature, usually by employing enzymes to provide the denaturing function of higher temperatures. For example, recombinase polymerase amplification (RPA) is a recently developed isothermal amplification technique (Piepenburg et al., 2006) that uses recombinase activity for DNA denaturation and strand displacement synthesis to amplify DNA targets. RPA uses two primers; the reaction runs between 37°C and 42°C and the results can be obtained in 10–30 min. The cyclic repetition of the process leads to exponential amplification (Ereku et al., 2018).

Helicase-dependent amplification (HDA) uses uvrD helicase of *Escherichia coli* instead of thermal denaturation to produce single-stranded DNA for primer annealing, and then primer extension occurs under isothermal conditions. The reaction occurs at 37°C and uses a reparation protein to activate uvrD helicase (Lau and Botella, 2017). Other techniques, such as nucleic acid sequence-based amplification (NASBA), can be used for amplification of either RNA or DNA sequences, although the technique is more used for RNA targets. NASBA is based on serial steps of transcription and reverse transcription processes. It uses a DNA oligonucleotide containing T7 promoter sequence at the 5′ end that anneals with target RNA and a T7 DNA polymerase. The reaction is conducted at 65°C, and the products are detected by quantification of fluorescently labeled probes (Ivanov et al., 2021).

In another approach, rolling-circle amplification (RCA) uses rolling replication of short, circular, single-stranded DNA molecules. This technique uses circularizing oligonucleotide probes, which are single-stranded DNA molecules that have target recognition sequences of 20 nucleotides present at both 5′ and 3′ ends. These oligonucleotide probes are hybridized to target regions and then become circularized using ligase. RCA requires a DNA polymerase, the component nucleotides, unwinding proteins, and primers with dual functions as signal amplifier and complementary sequence to the target DNA (Aslam et al., 2017).

Among these isothermal techniques, loop-mediated isothermal amplification (LAMP) is the best developed and most commonly applied for plant pathogen detection. A high number of LAMP-based approaches have been applied for detection of plant pathogens and numerous modifications that include portable devices, visualization techniques and standardization for multiple detection have been developed to improve its efficiency for detection of pathogens in the field. This is supported by more than 250 research articles on LAMP assays for detection of plant pathogens that have been published in the first decade following the original publication by Notomi et al. (2000) and a year-over-year increase in published LAMP research articles for plant pathogen detection since 2010 (Le and Vu, 2017). The focus of this review will be LAMP for detection of plant pathogens in wheat.

### LAMP Principle

LAMP is an alternative technique to PCR because it is time-efficient, labor-saving and shows a similar or better sensitivity and specificity compared with other RNA/DNA amplification methods (Panno et al., 2020). LAMP typically uses a set of six primers that are complementary to 8 regions in the target DNA coupled with a *Bst* DNA polymerase enzyme with strand displacement activity (Notomi et al., 2000; Nagamine et al., 2002). A primary advantage of LAMP is that it can be performed at a constant temperature between 60°C and 65°C, using only a simple water bath, and can be used even on crude template DNA or directly on tissue samples (Nagamine et al., 2002).

**Figure 1:**
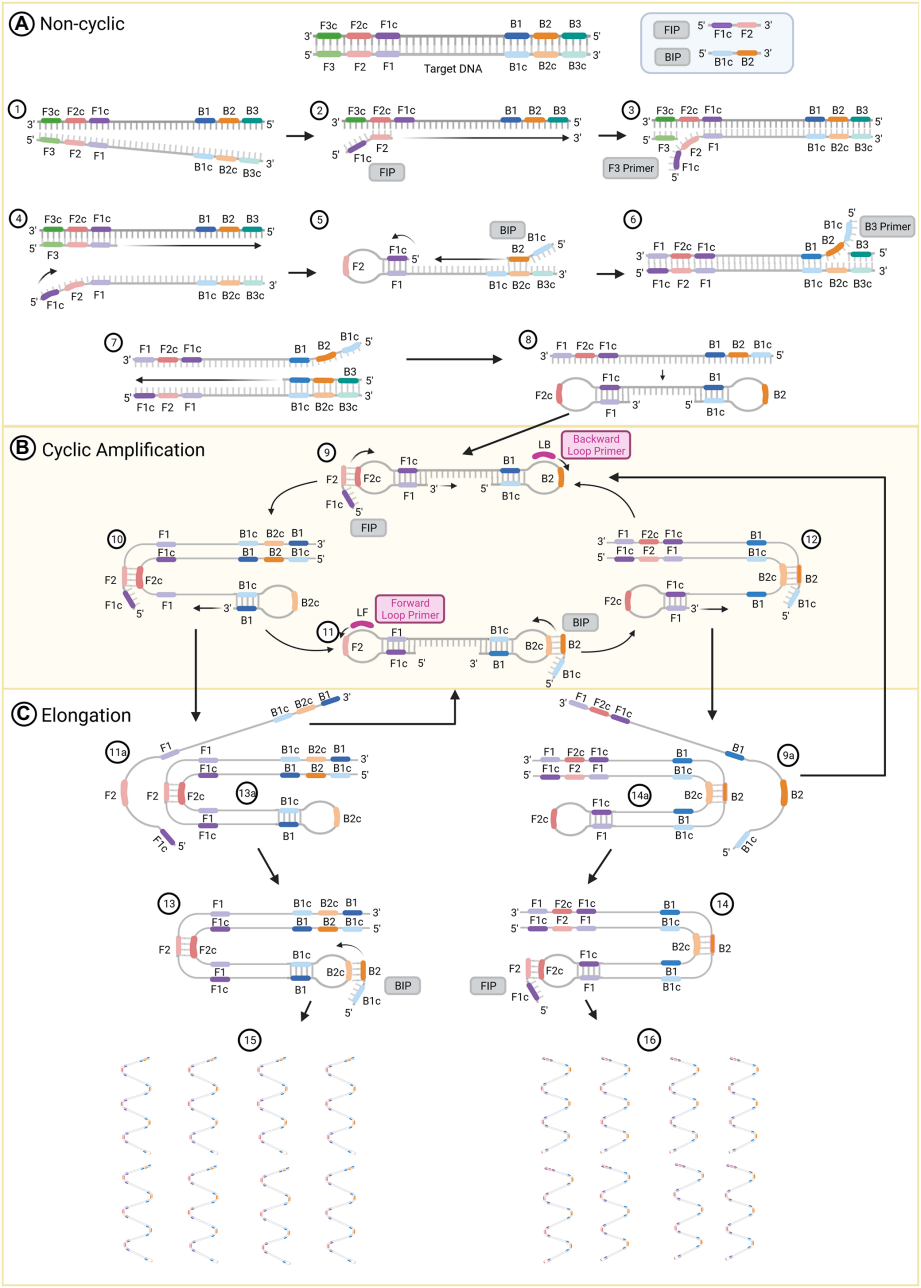
The loop-mediated isothermal amplification (LAMP) technique amplification process. **(A)** Non-cyclic steps that produce a DNA strand with two loops at their 5’ and 3’ ends. **(B)** Cyclic amplification steps and **(C)** elongation. Figure was created with BioRender.com.

LAMP generates DNA products with stem loop and cauliflower-like structures with multiple loops that result from auto-cycling, strand displacement DNA synthesis and the action of two inner primers, two outer primers and two optional loop primers (Iwamoto et al., 2003). The inner primers are used for priming the initial steps of the process (Figure 1, Steps 2 and 5) and for self-priming during the later stages of amplification (Figure 1, Steps 9 and 11). The first compound primer is called the forward inner primer (FIP), constituted by joining from 5′ to 3′ sequences designated F1c and F2. The second is designated the backward inner primer (BIP), constituted by joining sequences B2 and B1c (Notomi et al., 2000). Two outer primers only play a role during the non-cyclic step of strand displacement (Figure 1, Steps 3 and 6), and are denominated as the forward (F3) and backward (B3) outer primers (Parida et al., 2008). There are two loop primers that bind to additional sequences in the DNA that are not targeted by the four internal primers (Figure 1, Steps 9 and 11; Nagamine et al., 2002). Those are called the forward (LF) and backward (LB) loop primers; their role is to improve the amplification and accelerate the reaction (Parida et al., 2008; Duan et al., 2014b).

The reaction initiates with the FIP. The target DNA is denatured by heating to 95°C (Figure 1, Step 1) and rapidly cooled on ice. This permits primer invasion and annealing of the FIP to the target sequence (Notomi et al., 2000). The F2 region of the FIP anneals to the F2c section in the target DNA and initiates the synthesis of a complementary strand (Figure 1, Step 2). Use of a strand displacement polymerase allows this to occur at a constant temperature of 65°C. The outer primer F3 hybridizes to its complementary F3c sequence and initiates strand displacement DNA synthesis (Figure 1, Step 3), releasing a FIP-linked complementary strand (Figure 1, Step 4), which forms a loop structure at one end when the F1c half of the primer anneals to its F1 complementary sequence in the strand DNA (Figure 1, Step 5). This single-stranded DNA acts as a template for DNA synthesis initiated by the B2 sequence at the 3′ end of the BIP followed by B3-primed strand displacement DNA synthesis, which releases a BIP-linked complementary strand (Figure 1, Steps 5 to 7). This single-stranded DNA produces a dumb-bell structure, which is then converted to a stem-loop DNA by self-primed DNA synthesis (Figure 1, Step 8).

The stem-loop DNA produced during the non-cyclic part of the process provides the starting material for LAMP cycling, which is initiated by the FIP after annealing to the F2c sequence in the loop of the stem-loop DNA followed by primer strand displacement DNA synthesis (Figure 1, Steps 9 and 10). If added to the reaction, the LB primer binds to the loop between the B1 and B2 regions of the stem-loop DNA. This will generate additional stem-loop structures to which LB and LF primers can anneal to promote exponential amplification. At the same time, a strand displacement occurs from the 3′ end of the F1 region (Figure 1, Step 9), which opens the 5′ end loop (Figure 1, Step 10). Subsequently, a second strand displacement takes place from the 3′ end loop of the B1 region, which produces two intermediate structures (Steps 11a and 13a). One is a complementary double stem-loop DNA to the original one (Figure 1, Steps 11a to 11) and a new stem-loop DNA with a stem that is twice as long (Figure 1, Step 13a). These products provide the template for a BIP-primed strand displacement reaction in subsequent cycles (Figure 1, Step 13), which are called elongation and recycling. For the complementary double stem-loop DNA produced in steps 11a and 11, the subsequent DNA synthesis is initiated by the BIP through annealing to the loop in the stem-loop DNA and primer strand displacement DNA (Figure 1, Steps 11 and 12) in the same manner as for the original stem loop in step 9. If present, the LF primer binds to the loop between the F1 and F2 regions of this stem-loop structure and performs in the same manner as the LB primer to further accelerate LAMP. Two intermediate structures (Steps 9a and 14a) are produced from the structure in step 12. These structures are a double stem-loop DNA, which is the original one that was the starting material for LAMP cycling (Figure 1, Steps 9a and 9) and a new stem-loop DNA with a stem that is twice as long (Figure 1, Step 14a). These products will generate the template for a FIP-primed strand displacement reaction in the following cycles of elongation and recycling. Finally, more elongated structures (Figure 1, Steps 15 and 16) are produced. Through this process, the DNA target sequence is amplified 3-fold every half cycle (Notomi et al., 2000; Tomita et al., 2008; Bruce et al., 2015; Li and Macdonald, 2015).

Reverse transcription-LAMP (RT-LAMP) uses the Avian Myeloblastosis Virus (AMV) reverse transcriptase to amplify RNA target material which can perform the reaction in 60 min at a constant temperature in the same way as LAMP (Tomita et al., 2008).

### Visualization of Amplified Products

LAMP DNA amplicons can be visualized through gel electrophoresis or by adding post-amplification dyes to the solution. These methods require opening the tubes and involve a contamination risk due to the high amount of DNA that is produced during the reaction (Karthik et al., 2014; Fischbach et al., 2015). To fix that problem, a variety of visualization methods that are suitable for closed-tube amplification reactions can be used.

A closed-tube visual inspection uses DNA-intercalating dyes, such as SYBR Green, EvaGreen, ethidium bromide, propidium iodide, and Quanti-iT PicoGreen, or Hydroxynaphthol blue (HNB; Duan et al., 2014a), calcein (Zhou et al., 2014), or CuSO_4_ (Tomita et al., 2008), which are metal ion indicators (Zhang et al., 2013; Panno et al., 2020).

SYBR Green has been shown to inhibit real-time LAMP reactions when it is present at concentrations of 1–5 μM or when 0.5 mM Mn^2+^ is added to the pre-reaction solution (Goto et al., 2009; Abbasi et al., 2016). To fix this problem, SYBR Green has been replaced with SYTO-16 stain for real-time LAMP or with metal indicators (HNB and calcein) in common LAMP reactions. HNB shows a very good performance in product visualization. Its detection sensitivity is equivalent to SYBR green and it can be present during the reaction, which decreases cross-contamination (Goto et al., 2009). Calcein and HNB can identify amplified products by detecting the change of metal ion concentration in the solution during LAMP. In this process, pyrophosphate ions are produced in great amounts and form insoluble salts by binding with metal ions, such as manganese. As a result, the manganese concentration decreases and the manganese ions that were previously combined with calcein for the reaction to quench, make the solution appear orange. Once LAMP starts in the presence of DNA, calcein is deprived of manganese ions by the new pyrophosphate ion that is generated and calcein can combine with residual magnesium, producing fluorescence (Tomita et al., 2008).

Detection of LAMP products also can be conducted through visual observation of turbidity in the solution, or with a photometer for quantitative detection. The latter is a real-time process that monitors the increase in DNA amplicons by measuring Mg^2+^ ion concentration in the solution (Parida et al., 2005; Goto et al., 2009; Zhang et al., 2013).

### Quantitative LAMP

Post-amplification methods to measure LAMP products in end-point analysis can lead to cross-contamination, false positives, or non-specific detection of amplicons. These methods include agarose gel electrophoresis, turbidity observation, detection using intercalating dyes and addition of metal ions as indicators (Gadkar et al., 2018). The development of quantitative or real-time LAMP reduces potential errors from post-amplification detection, enables quantitative detection of the amplified products and is more convenient for multi-sample analysis.

The most widely used real-time LAMP methods are based on measuring the turbidity of the solution or on measurement of fluorescence emission by intercalating dyes, such as ethidium bromide or SYBR Green I (Panno et al., 2020). More recently, the use of fluorescent assimilating probes has optimized real-time LAMP and is a solution to non-specific problems of dye-based detection systems (Villari et al., 2017; Gadkar et al., 2018). The real-time turbidity approach quantifies the amount of magnesium pyrophosphate produced as a byproduct of the LAMP reaction using a real-time turbidimeter. A commercial real-time turbidimeter device (Eiken Chemical, Co., Ltd., Japan) was developed by Mori et al. (2004) and is currently available for quantitative LAMP. The device can maintain the LAMP reaction at an optimum temperature (60°C–65°C) and measures the turbidity of multiple samples simultaneously (Mori et al., 2004; Panno et al., 2020). Regardless of the utility of the turbidity-based detection method, it is 10 times less sensitive than real-time LAMP using fluorescent probes (Quyen et al., 2019).

The fluorescence resonance energy transfer (FRET) approach uses a pair of labeled oligonucleotide probes to optimize LAMP for quantitative detection, in which a quenching strand is displaced from a partially complementary fluorescent strand during the DNA synthesis process. Several portable devices are commercially available to apply this technology, such as “Bioranger” (Diagenetix, Inc., Honolulu, HI, United States) and “Genie II and III” (Optigene Ltd., West Sussex, United Kingdom; Thiessen et al., 2018), Additionally, quantification of LAMP products using a fluorescent dye can be performed using a real-time PCR thermal cycler. A portable fluorescence reader called the ESE-Quant Tube Scanner is also used and offers a convenient alternative for rapid on-site detection (Cao et al., 2017). Recently, this method was used for detection of *Ustilago maydis* in infested soil samples and maize plants using the effector genes *Pep1*, *Pit2*, and *See1* as targets and the Bio-Rad CFX96 Real-Time PCR system to measure the fluorescence signal (Cao et al., 2017). For detection of pathogens in wheat, real-time LAMP has been used for quantification and identification of Wheat Dwarf Virus (Trzmiel and Hasiów-Jaroszewska, 2020; Hao et al., 2021), *Pyricularia oryzae* Triticum lineage (Yasuhara-Bell et al., 2018), *Tilletia* species (Pieczul et al., 2018), *F. graminearum* (Gupta et al., 2020) and trichothecene mycotoxins produced by *Fusarium* species (Denschlag et al., 2014) as will be described later. A quantitative, assimilating probe-based LAMP has been applied for airborne inoculum detection of *Magnaporthe oryzae*, the causal agent of gray leaf spot in turfgrass fields. They combined this approach with the use of a spore trap system and tested its suitability for implementation in the field (Villari et al., 2017).

### Multiplex LAMP

Traditional techniques for detection of LAMP products are only useful for a single target sequence in the same reaction. Multiplex LAMP (mLAMP) can discriminate target-specific amplicons from a mixture of LAMP products. It is commonly performed by introducing an endonuclease recognition site into the LAMP primers which allows generation of endonuclease-digested amplicons with a length that is specific to the target species (Liang et al., 2012).

mLAMP can use sequence-based barcodes coupled with nicking endonuclease-mediated pyrosequencing (Liang et al., 2012). In this method, a short sequence in the middle of a FIP is used as a target-specific barcode. A recognition site for nicking endonuclease (NEase) is introduced into the FIP. After LAMP reaction, this recognition site allows the use of pyrosequencing, a sequence-by-synthesis method, to decode the barcodes. Because NEases only cleave one specific strand of a duplex DNA, the 3′ end at the nick is extendable by *Bst* polymerase, which has strand displacement activity. NEase-digested LAMP products then can be pyrosequenced directly without the use of any primer annealing process (Liang et al., 2012).

Another technique, the multiple endonuclease restriction real-time (MERT)-LAMP assay, combines endonuclease restriction and real-time fluorescence detection with LAMP (Wang et al., 2015). The real-time mLAMP technique combines a standard real-time fluorimeter with the mLAMP assay and can detect 1–4 target sequences in a single reaction tube (Tanner et al., 2012). This technique uses LAMP primers with a quencher–fluorophore duplex region, which displays a fluorescent signal after strand separation (Panno et al., 2020).

mLAMP has been coupled with a variety of multiplex product detection methods to differentiate each amplicon in the products. This technique has been combined with dot-ELISA (Nkouawa et al., 2016), and a LAMP-PCR in combination with hybridization, digestion with restriction endonuclease and the colorimetric method of ELISA has also been applied in medical studies (Lee et al., 2010).

A mLAMP assay was applied to detect the *P. oryzae* Lolium and Triticum lineages in wheat. The mitochondrial NADH-dehydrogenase (*nad5*) gene was used as an internal control for plant DNA, and it was multiplexed with the Pot2 and MoT3 genes (Yasuhara-Bell et al., 2018). This assay was conducted to confirm results from a previous LAMP assay and to compare the sensitivity of mLAMP for rapid detection of *P. oryzae* pathotypes. The Nad5/PoT2 mLAMP assay was first applied to detect *P. oryzae* in the samples. Then, the Nad5/MoT3 assay was applied to confirm whether the identified *P. oryzae* strain corresponded to the *P. oryzae* Triticum lineage. The mLAMP assay results were measured using a CFX96 Real-Time System (Bio-Rad, Hercules, CA, United States) with fluorescence reading at 1-min intervals. The results obtained in the mLAMP assay were comparable to those of the individual analysis (Yasuhara-Bell et al., 2018). Multiplex LAMP techniques do not appear to have been developed for other pathogens of wheat.

### Other LAMP Approaches

LAMP assay can be combined with microfluidic technology to miniaturize the LAMP detection system and facilitate point-of-care pathogen detection. This was motivated by developments in microfluidics technology that allow the manipulation of small volumes of liquids in microfabricated channels or in microchannels to perform all analytical steps (Fang et al., 2010). LAMP is integrated on a microfluidic chip either for readout by eye or for measurement by an optic sensor, which allows detection of target nucleic acids and maintains the sensitivity, rapidity, and specificity of LAMP. This technique is called μLAMP and requires a small sample volume of 0.4 μl. This integrated approach has great potential to make LAMP highly portable for on-site analyses (Panno et al., 2020).

Digital LAMP (dLAMP) is an approach that allows accurate quantification of DNA or RNA in a target sample. The total sample is distributed into small compartments such that each compartment contains approximately less than one template molecule (Gansen et al., 2012; Panno et al., 2020). DNA amplification is conducted in each compartment and the number of initial templates in the original sample is equivalent to the number of compartments that show amplification. A sample self-digitization (SD) chip was developed to provide a simple, inexpensive, and sample-conserving device with self-consistent nanoliter compartments and straightforward chip operation. This device is robust and can automatically digitize a sample into an array of nanoliter wells without losing any sample volume. This is called a self-digitization of the total sample. These nanoliter individual volumes will later be used in dLAMP (Gansen et al., 2012).

Electric LAMP (eLAMP) is conducted through an electronic simulation that performs putative tests of LAMP primers on target sequences to determine compatibility. eLAMP can be used to test previously available sets of primers to detect recently discovered sequence variants (Wong et al., 2018). In-disc LAMP (iD-LAMP) is based on the “lab-on-a-disc” concept, in which genomic assays are performed in centrifugal devices that integrate all the analytical steps by controlling the rotation rate. iD-LAMP integrates LAMP amplification and compact disc technology, using an integrated device composed of micro-reactors embedded onto CDs for real-time targeted DNA determination. The real-time curves are measured by cyclic scanning using the optics of a DVD drive and the measurement is taken with standard instruments, such as colorimeters or fluorescence microscopes (Santiago-Felipe et al., 2016).

LAMP can be coupled with a Lateral Flow Dipstick (LFD) device, which can detect biotin-labeled amplicons upon hybridization to a fluorescein-labeled DNA probe complexed with a gold-labeled anti-fluorescein antibody (Rigano et al., 2014; Ivanov et al., 2021). LAMP reaction is carried out for 30 min at 65°C, then a specific fluorescence-labeled probe is added to the reaction mixture and incubated for another 10 min. The LFD strip is inserted into the tube. The resulting complex moves by capillarity and is trapped by a biotin ligand at the test zone. The local gold concentration increases, and the positive reaction can be observed as a reddish-brown color line that develops on the test zone (Ivanov et al., 2021). This has the potential to replace visualization methods that are not compatible with field applications, such as gel electrophoresis (Rigano et al., 2014).

Other approaches, such as lyophilized LAMP, aim to simplify the reaction process by providing a lyophilized LAMP mix that contains all the reagents (Le and Vu, 2017). The incubation is performed after adding water and sample or DNA–RNA template into the lyophilized mix. There are some lyophilized LAMP kits that are commercially available associated with portable thermal cyclers and other devices, which make it suitable under field conditions (Panno et al., 2020).

## LAMP for Detection of Wheat Pathogens

### ***Fusarium*** Species

*Fusarium graminearum*, the causal agent of Fusarium head blight (FHB), was detected using LAMP in an experiment that also tested DNA from 177 strains from 21 genera of filamentous fungi and two genera of yeast. The primers were designed from a 2042 bp fragment of the *gaoA* gene (galactose oxidase precursor) from *F. austroamericanum* isolate NRRL 2903, and the LAMP technique was applied directly to barley grains and wheat seeds. The *gaoA* gene was selected as a target because *F. graminearum* is the only species showing galactose oxidase activity in culture supernatants (Niessen and Vogel, 2010). Calcein fluorescence was observed with DNA from all *F. graminearum* isolates and in strains representing very similar lineages, such as those in section *Discolor*, which presumably possess sequences homologous to the *gaoA* gene. However, when they tested a species that is closely related to *F. graminearum*, only the target species gave fluorescence signals, which confirmed the specificity of these primers and the LAMP technique. Sensitivity was also confirmed by obtaining fluorescence and amplified products of a predicted size of 145 bp with DNA concentrations below 2 pg/reaction (Niessen and Vogel, 2010).

Traditional and real-time duplex LAMP reactions were conducted to detect deoxynivalenol (DON), nivalenol (NIV), and T2-Toxin, which are trichothecene mycotoxins produced by *Fusarium* species on cereals. Target genes for LAMP reactions were *tri6* (657-bp coding sequence of a regulatory protein) from *F. graminearum* and *tri5* (694-bp trichodiene synthase coding sequence) from *F. sporotrichioides*. The LAMP reaction was performed on 100 wheat samples and 127 fungal species that were used as controls to confirm the specificity of the technique to detect only the target trichothecene-producing *Fusarium* spp. A real-time turbidimeter was used for incubation and the optimum temperature to obtain DNA amplification was 64°C for both primer sets *Tri6* and *Tri5*. The LAMP assay produced DNA amplicons if DON concentration was greater than 162 ppb in the samples. When both sets of primers were used in the duplex assay, it was possible to detect *F. graminearum*, *F. culmorum*, *F. cerealis*, *F. sporotrichioides*, *F. langsethiae*, and *F. poae* in a group-specific manner. This means that the whole group of potentially trichothecene-producing *Fusarium* spp. was detected with this assay. The LAMP assay was able to detect amplified products for the species between DNA concentrations of 0.004 ng for *F. graminearum* and 15.74 ng for *F. poae* (Denschlag et al., 2014).

### Pyricularia oryzae

Wheat blast caused by *P. oryzae* Triticum lineage shows symptoms similar to those seen with FHB. High specificity was achieved in identifying the *P. oryzae* Triticum lineage using LAMP on 158 strains of *P. oryzae* and 50 strains of *F. graminearum* (Yasuhara-Bell et al., 2018). The primers used for this experiment were designed to target the PoT2 locus (Harmon et al., 2003), which differentiates *P. oryzae* from other genera of fungi, and the MoT3 locus to differentiate between pathotypes. Identification of *P. oryzae* was achieved with a minimum amount of 5 pg/μl of DNA per reaction, which indicates high detection sensitivity. The detection was performed using a portable and robust instrument for isothermal amplification called the Genie II system (Yasuhara-Bell et al., 2018).

LAMP, along with PCR and qPCR were used to develop a toolkit of detection tests that can improve current wheat blast detection. The ability of these tests to detect the *P. oryzae* Triticum lineage on contaminated wheat grains was evaluated. Five groups of primers were designed for LAMP and were applied on three wheat-derived isolates and four non-wheat-derived isolates (Thierry et al., 2020). The primers targeted polymorphisms located in genomic regions to find a detection method with improved specificity for the Triticum lineage of *P. oryzae*. One group of primers amplified DNA from every wheat-derived isolate in a very short time, although full specificity was not achieved. However, high sensitivity was obtained with these primers when used on dilutions of down to 5 pg of genomic DNA of three isolates. LAMP failed to amplify DNA of *P oryzae* from contaminated seed lots when no incubation of the seeds in potato dextrose broth was conducted. However, when this incubation step was included, the detection improved in all tests and LAMP primers were able to detect the pathogen for all replicates in less than 5 min. PCR did not amplify *P. oryzae* isolated from other species in the Poaceae which demonstrated a higher level of specificity. LAMP was suggested as a quick pre-screening test that can provide results within 8 min; a posterior confirmation of positive results should be done by PCR or qPCR (Thierry et al., 2020).

### *Puccinia* Species

*Puccinia striiformis* f. sp. *tritici* is the causal agent of wheat stripe (or yellow) rust. The specificity of LAMP was tested to identify DNA from *P. striiformis* using four isolates of this pathogen and DNA samples from the related rust fungi *P. graminis* f. sp. *tritici* (the cause of stem rust) and *P. recondita* f. sp. *tritici* (synonym *P. triticina*, leaf rust), plus the unrelated wheat pathogens *Alternaria triticina* (leaf blight), *Blumeria graminis* f. sp. *tritici* (powdery mildew), *Bipolaris sorokiniana* (*Cochliobolus sativus*, spot blotch, foot, and root rot), *Fusarium graminearum* (FHB), and *Rhizoctonia cerealis* (sharp eyespot). LAMP primers were designed from *β-tubulin* gene sequence. *P. striiformis* was detected using SYBR Green I and the amplified product showed bands of the expected size, while no bands were observed for any other fungal pathogens including close relatives. Sensitivity was confirmed with DNA samples from spores, which were amplified from a concentration of 2 pg/μl. Even higher sensitivity was obtained with DNA from inoculated wheat leaves (Huang et al., 2011).

The accuracy and specificity of LAMP for detection of *P. striiformis* f. sp. *tritici* was confirmed with DNA from urediniospores and wheat seedlings with latent infections. The wheat pathogens *Bipolaris sorokiniana*, *Blumeria graminis*, *Fusarium graminearum*, and *Tilletia indica* (Karnal bunt), plus the additional fungi *Aspergillus niger*, *Bipolaris oryzae*, and *Rhizoctonia solani* were used as negative controls to confirm specific detection of *P. striiformis* f. sp. *tritici*. Primers were designed from a qPCR-based marker developed from the ketopantoate reductase coding sequence present in the genome. This gene has been used to analyze the evolutionary relationships among *P. striiformis* f. sp. *tritici* pathotypes (Aggarwal et al., 2018). DNA fragments were detected through the use of HNB dye and ethidium bromide reagent, and ladder-like DNA fragments were amplified with up to 1 pg/μl of DNA concentration, being 10-fold more sensitive than conventional PCR. LAMP also produced an accurate detection with field samples under optimized conditions. Together, these results showed that LAMP has very high sensitivity for detection of *P. striiformis* f. sp. *tritici* and can be applied directly to field samples (Aggarwal et al., 2017).

### Smut Pathogens

LAMP was used to detect three species of smut fungi that cause common bunt and dwarf bunt, which are important seedborne diseases in wheat. DNA samples from wheat grains infected with teliospores from *Tilletia caries* (common bunt), *T. controversa* (dwarf bunt), and *T. laevis* (common bunt, smooth-spored wheat bunt, stinking smut) were used in a LAMP reaction to test for accurate detection of these pathogens. Other common fungal species in wheat grain (*Alternaria alternata*, *Cladosporium cladosporioides*, *Fusarium avenaceum*, *F. culmorum*, *F. graminearum*, *F. poae*, *Helminthosporium* sp., and *Penicillium* sp.) were also subjected to the assay to determine LAMP specificity. Negative results were obtained for all the tested isolates of the non-smut fungal species plus a control that contained water with the reagents but no DNA. Amplification was obtained for *T. caries* and *T. laevis*, with a detection limit in wheat grain of 20 ug of teliospores per 100 g of grain, while *T. controversa* had a detection limit of 20 mg of teliospores per 100 g of grain. The minimum DNA concentration that LAMP was able to detect for the three smut species was around 0.001 ng/μl (Pieczul et al., 2018).

*Ustilago tritici*, causal agent of loose smut of wheat, also was detected using LAMP. The amplification technique showed a detection limit of 100 fg/μl of DNA, which was 100 times lower than that obtained with qPCR (10 pg/μl). Primers for LAMP were designed to target the large ribosomal subunit gene and the ITS region. DNA samples from the wheat pathogens *Bipolaris sorokiniana*, *Blumeria graminis*, *F. graminearum*, *P. striiformis*, and *R. cerealis*, plus the potato and tomato pathogen *Alternaria solani* were used as negative controls to test LAMP specificity for detection of *U. tritici* by confirming the non-amplification of DNA from samples of these pathogens. These pathogens were used as controls because there are previous reports on the detection of some of them by qPCR analysis and the detection of Fusarium head blight and wheat stripe rust by LAMP assays. No amplification was obtained with the designed primers on the negative controls. This result was confirmed through fluorescence detection using SYBR Green I. The optimum reaction temperature for detection of *U. tritici* was 63°C (Yan et al., 2019).

### Viruses

*Wheat yellow mosaic virus* (WYMV) was detected using reverse transcription, loop-mediated isothermal amplification (RT-LAMP). Four primer sets, designed to target the coat protein of the virus, were used to perform the reaction. The specificity of the reaction was tested with two wheat viruses [Chinese Wheat Mosaic Virus (CWMV) and Barley Stripe Mosaic Virus (BSMV)], and the negative control was RNA collected from healthy wheat. Total RNA from wheat leaves infected with each virus was extracted and used for the reaction. They found that 65°C for 80 min were the optimal temperature and time to obtain DNA amplicons, although they could detect the virus after 30 or 45 min. DNA amplicons were visualized through observation of turbidity in the solution and agarose gel electrophoresis. Amplification was obtained only for WYMV, and no DNA amplicons were observed for CWMV or BSMV. The RT-LAMP technique was 100 times more sensitive than RT-PCR and detected RNA that was diluted up to 10^−5^ (Zhang et al., 2011).

### Other Pathogens of Wheat

Despite the importance of wheat leaf blotch-pathogenic fungi, such as *Z. tritici*, *Parastagonospora nodorum*, *Pyrenophora tritici-repentis*, and *B. sorokiniana*, no LAMP assay has yet been reported for detection of these pathogens in the field. However, a LAMP assay was reported for specific detection of fungicide resistance in *Z. tritici*, using two promoter inserts in the *MgCYP51* and *MgMFS1* genes as a target, which are associated with gene overexpression and increased fungicide efflux in this fungus (King et al., 2016). This LAMP assay was validated through its application on a variety of *Z. tritici* isolates, in which PCR was also applied for confirmation. They concluded that the LAMP assay can be used to detect geographical spread of these promoter inserts in *Z. tritici* strains and can be a useful tool for Septoria tritici blotch management and to minimize fungicide resistance (King et al., 2016). The leaf blotch diseases caused by all of these fungi can co-occur and often are difficult to diagnose, so a LAMP assay to identify and detect these pathogens should be a high priority for future research. A summary of research on the detection of wheat pathogens using LAMP is provided in Table 1 and a summary of research on the detection of wheat pathogens using other isothermal-based techniques is provided in Table 2.

**Table 1:** LAMP-based detection of various pathogens in wheat.

Pathogen	Disease	Target gene	Visualization technique	References
*Wheat dwarf virus* (WDV)	Wheat dwarf	Coat protein	Gel electrophoresis, real-time monitoring of amplification curves, SYBRGreen I dye	Trzmiel and Hasiów-Jaroszewska, 2020
*Wheat streak mosaic virus* (WSMV)	Wheat streak mosaic	Poly-protein coding gene	Electrophoresis in agarose gel	Lee et al., 2015
*Wheat yellow mosaic virus* (WYMV)	Wheat yellow mosaic	Coat protein	Turbidity observation and electrophoresis	Zhang et al., 2011
*Pyricularia oryzae* Triticum lineage	Wheat blast	PoT2 and MoT3 loci	Real-time fluorescence and Genie II system	Yasuhara-Bell et al., 2018
*Puccinia triticina* (synonym: *P. recondita* f. sp. *tritici*)	Leaf rust	*PtRA_68_* specific marker	Hydroxy naphthol Blue (HNB) visualizing indicator; electrophoresis in agarose gel	Manjunatha et al., 2018
*Puccinia striiformis* f. sp. *tritici*	Wheat stripe (or yellow) rust	*β-tubulin* gene	SYBR Green I and electrophoresis in agarose gel	Huang et al., 2011
		Ketopantoate reductase coding sequence	HNB dye and ethidium bromide, electrophoresis	Aggarwal et al., 2017
*Tilletia caries*, *T. controversa*, and *T. laevis*	Common bunt, dwarf bunt and smooth-spored wheat bunt	IGS 2 rDNA	Real-time monitoring with melting curves, electrophoresis, and direct fluorescence	Pieczul et al., 2018
*Ustilago tritici*	Loose smut of wheat	Large ribosomal unit and ITS region	SYBR Green I	Yan et al., 2019
*Fusarium asiaticum*	Fusarium head blight	*CYP51C* gene	Hydroxy naphthol Blue (HNB) visualizing indicator	Xu et al., 2017
*Fusarium graminearum*	Fusarium head blight	218-bp region from a partial sequence of *F. graminearum* chromosome 1	Hydroxy naphthol Blue (HNB) visualizing indicator; electrophoresis in agarose gel	Gupta et al., 2020
		*gaoA* gene (galactose oxidase precursor)	Real-time calcein fluorescence; electrophoresis in agarose gel	Niessen and Vogel, 2010
*Fusarium* species	Deoxynivalenol (DON), nivalenol (NIV) and T2-Toxin	*tri5* gene (trichodiene synthase) and *tri6* (biosynthesis of trichothecenes)	Real-time turbidimeter amplification curves	Denschlag et al., 2014
*Zymoseptoria tritici* (fungicide resistance)	Septoria tritici blotch	*MgCYP51* and *MgMFS1* genes	Gel electrophoresis	King et al., 2016

**Table 2:** Isothermal-based detection of various pathogens in wheat.

Isothermal-based technique	Pathogen	Disease	Target gene	References
RPA	*Bipolaris sorokiniana*	Root rot and spot blotch	Calmodulin (*cal*)	Zhao et al., 2021
RPA	*Wheat dwarf virus* (WDV)	Wheat dwarf	Polymorphic 12 nucleotides motif (nt 1,433–1,444)	Glais and Jacquot, 2015
RT-RPA	*Barley yellow dwarf virus* (BYDV)	Yellow dwarf of wheat	Coat protein (CP) gene	Kim et al., 2020
RCA	*Fusarium graminearum* species complex (FGSC)	Fusarium Head Blight (FHB)	Elongation factor 1-α (EF-1α)	Davari et al., 2012
RT-HDA	*High plains virus* (HPV)	High plains of wheat	Nucleoprotein gene	Arif et al., 2014

*RPA, recombinase polymerase amplification; RT-RPA, reverse transcription RPA; RCA, rolling-circle amplification; and RT-HDA, reverse transcription helicase-dependent amplification*.

## Discussion

The most significant features of LAMP are the constant temperature conditions, that avoid the use of a thermal cycler, and the rapidity of the reaction, which can be completed in about an hour, or in less than 30 min if loop primers are used. LAMP shows high specificity and sensitivity due to the use of six primers that can target eight regions in the DNA. The technique can be applied either on purified DNA samples or directly in infected wheat tissues, which reduces the detection time and the equipment required. LAMP is a versatile molecular technique due to the variety of visualization methods and to modifications of the original LAMP procedure, which have given rise to RT-LAMP (Reverse transcriptase—Loop-Mediated Isothermal Amplification), real-time LAMP, and multiplex LAMP. These methods have shown specificity and sensitivity levels similar to or better than those of PCR techniques. The efficiency of RT-LAMP is due to the rapid amplification provided by the loop structure and strand displacement polymerase, plus the robustness of the enzymes used for this methodology, which minimizes inhibitor problems.

Real-time LAMP assays have been applied for portable detection of plant pathogens in other crops and can be an alternative for applications in wheat. These assays have applied real-time LAMP using the portable instrument Genie ® II (Aglietti et al., 2019). This opens a new perspective for use of portable devices that apply the LAMP technique in the field. Genie II and Genie III are small, low-maintenance, and portable devices. These instruments are capable of temperature control up to 100°C and simultaneous fluorescence detection *via* the FAM channel. Genie II contains two blocks with eight samples in each block, while Genie III includes a single block that accommodates eight samples (Domesle et al., 2020). A LAMP assay with a portable fluorometer (Genelyzer III) using a toothpick method has also been used for detection of plant pathogens (Wilisiani et al., 2019).

Recently, a LAMP-based foldable microdevice platform based on fuchsin colorimetric detection was developed to detect *P. oryzae* and *Sarocladium oryzae* in rice seeds, but this approach will require standardization before its application to other pathogen species (Prasannakumar et al., 2021). Other examples of portable devices for detecting LAMP products include the ESE-Quant tube scanner (Qiagen, Netherlands) and the Bio-Rad CFX96 Real-Time PCR system that were used for portable real-time LAMP and fluorescence measurement for detection of *Ustilago maydis* (Cao et al., 2017). A POCKET (point-of-care kit for the entire test) platform was developed that can be coupled with isothermal amplification techniques (Xu et al., 2020). This device is ultraportable and uses a smartphone as a heater to maintain an isothermal incubation, and as a signal detector and result readout. Additionally, a commercial membrane instead of a chip to conduct dLAMP was developed to be applied for point-of-care detection (Lin et al., 2019). The membrane is made of track-etched polycarbonate and each pore within the membrane functions as an individual nanoreactor for single DNA amplification. The new method is portable and possibly the most inexpensive way to perform dLAMP (Lin et al., 2019). A summary of recent LAMP-based approaches for detection of pathogens in plants other than wheat is provided in Table 3.

**Table 3:** Recent LAMP-based approaches for detection of pathogens in other plants.

LAMP-based approach	Pathogen	Disease	Target sequence	References
Multiplex RT-LAMP	Banana bunchy top virus (BBTV), banana streak viruses (BSVs), cucumber mosaic virus (CMV)	Banana bunchy top, banana streak, cucumber mosaic	Conserved regions of coat protein sequence	Zhang et al., 2018
Portable LAMP. Genie II instrument	*Neofabraea perennans*	Bull’s eye rot (BER) in apple and pear	*β-tubulin* gene	Enicks et al., 2020
LAMP-Coupled CRISPR-Cas12a module	Tomato yellow leaf curl virus (TYLCV) and Tomato leaf curl New Delhi virus (ToLCNDV)	Tomato yellow leaf curl and Tomato leaf curl New Delhi	Coat protein gene (CP)	Mahas et al., 2021
Real-time colorimetric LAMP	*Xanthomonas gardneri*	Bacterial spot (BS) of tomato and pepper	*hrpB* gene	Stehlíková et al., 2020
Probe-based real-time LAMP	*L. acicola*, *D. pini* and *D. septosporum*	Spot needle blight (BSNB) and *Dothistroma* needle blight (DNB)	Elongation factor (*EF1- α*) and beta-tubulin (*β-tub2*)	Aglietti et al., 2021
FRET-based probe qLAMP	*Erysiphe necator*	Grape powdery mildew	ITS region	Thiessen et al., 2018
LAMP-based Turn-on Fluorescent Paper (ToFP)	*Rosellinia necatrix*	White root rot (WRR)	Template candidates from regions in strain W97, scaffold, contig 1 sequence	Lee et al., 2020
Microneedle-smartphone-based LAMP and RT-LAMP	*Phytophthora infestans* and Tomato spotted wilt virus (TSWV)	Late blight on potato and tomato, and tomato spotted wilt	ITS sequence in *P. infestans* and N gene of TSWV.	Paul et al., 2021
Cas12a PAM-free LAMP (Cas-PfLAMP)	*Xanthomonas oryzae* pv. Oryzae, rice stripe virus (RSV), and rice black-streaked dwarf virus (RBSDV)	Rice bacterial leaf blight, rice stripe, rice black-straked dwarf	*PilV* gene from *X. oryzae* pv. Oryzae, RSV SD-JN2 *RNA4* segment, RBSDV N89 *P1* gene.	Zhu et al., 2022

Limitations of the LAMP technique include a high risk of cross-contamination and subsequent false-positive results in controls, because of its high efficiency in DNA amplification. Additional caution is required for open-tube visualization to avoid cross-contamination (Le and Vu, 2017). Use of multiple primers also increases the chances of dimer formation and primer–primer hybridizations, which can give unreliable results (Wong et al., 2018). Designing the primers used in LAMP can be a complicated and non-intuitive process, which makes it difficult for those who are not experts (Lau and Botella, 2017). However, a LAMP primer tool exists (Primer Explorer V5) and is available online, which includes tutorials and a step-by-step guide for primer design (Notomi et al., 2015). Also, the design and use of six primers in LAMP, although more challenging, provides very high specificity and sensitivity.

Compared to PCR, LAMP may not be as cost-effective for reagents because it requires the use of multiple primer sets and *Bst* polymerase. However, LAMP only needs a water bath or a block heater, which shows its applicability in a resource-limited context and is cheaper than a dedicated PCR machine. Also, LAMP saves time and labor because the reaction is rapid and can be performed by non-specialized personnel (Panno et al., 2020). For LAMP applications in the field, the temperature required (60°C–65°C) can be a limitation. A common block heater or water bath can be used, but these tools require electricity which may not be available in the field. This can be overcome with an electricity-free heater based on exothermic chemical reactions and engineered phase change materials that is suitable for any kind of isothermal amplification technique (LaBarre et al., 2011; Panno et al., 2020). In addition, a device, such as POCKET that uses smartphone technology as a heater for isothermal reactions, is a great alternative to convert LAMP into an efficient and convenient field assay (Xu et al., 2020).

Simultaneous detection of multiple plant pathogens in wheat is required to achieve early discrimination of the causal agent and rapid application of management techniques. Parallelized LAMP and mLAMP are two alternatives that can be applied for this purpose. The first one can be performed using microfluidic diagnostic or lab-on-a-chip devices (Zhao et al., 2019). Microfluidic devices integrate a network of microchannels, in which individual samples and different sets of LAMP primers can be added for specific detection of target pathogens (Natsuhara et al., 2020). Parallel LAMP using this technology requires lower consumption of reagents than mLAMP and can increase the portability of the technique, allowing on-site detection without expert knowledge and skills (Zhao et al., 2019). A drawback is the cost of some lab-on-a-chip devices, which require the use of unique and sophisticated equipment for their manufacture or signal interpretation. To date, most existing microfluidic systems are complex and expensive to integrate into a functional system (Zhao et al., 2019; Ivanov et al., 2021).

Different techniques have been developed and tested to improve mLAMP for pathogen detection, such as portable fluorescence devices, multiple endonuclease restriction real-time (MERT)-LAMP, and mLAMP coupled with dot-ELISA (Wang et al., 2015; Nkouawa et al., 2016). These showed promising results in detection of pathogens and can be alternatives for applications in plant pathogen detection, although they can be time-consuming and require expensive sequencing equipment and reagents (Wang et al., 2015). mLAMP assays applied to detect numerous species also need to be designed carefully to avoid interference or non-desired interactions between primers. In contrast, microfluidic technology for parallelized LAMP allows for detection of up to 1,200 samples simultaneously while maintaining sensitivity (Oliveira et al., 2021). Some mLAMP techniques have shown great potential to be applied for detection of wheat pathogens (Yasuhara-Bell et al., 2018; Kang et al., 2020), while parallelized LAMP using microfluidics is starting to become popular for plant pathogen detection with some examples involving plant viruses (Natsuhara et al., 2020).

Application of LAMP-based approaches to detect pathogens in wheat will require some modifications and factors that must be taken into consideration. First, the design of primer sets must allow for specific detection of each species in the pathogen complex that affect this crop. The uniqueness of the selected target sequences in each species must be validated to ensure no similarity with other pathogen species of wheat is found (Manjunatha et al., 2018). This is highly relevant for the wheat pathogen complex, in which some species are closely related and produce very similar symptoms.

Special attention should be given to diseases, such as wheat blast, which is caused by different isolates belonging to the Triticum lineage. In this case, the LAMP technique must be able to discriminate lineages responsible for wheat blast epidemics from those belonging to the other host-specific lineages of the species, which do not incite blast but may be capable of causing opportunistic infections on wheat plants (Thierry et al., 2020). For this purpose, primers have been designed that target new genomic regions to identify polymorphisms fully specific to the *Triticum* lineage (Thierry et al., 2020).

Co-occurrence of pathogen species in wheat is common and is thought to have important implications for pathogen ecology and evolution, as well as for management techniques (Abdullah et al., 2018). Co-infections in wheat are caused by pathogens from different lifestyles and modes of nutrition, which may impact the selection of management techniques. Detection and quantification of the predominant causal agent provides useful information to direct early strategies for control. Accurate detection of the dominant causal agent in wheat can be achieved by applying qLAMP coupled with mLAMP. For this purpose, the MERT-LAMP assay (Wang et al., 2015) is a promising technique for application in wheat that is able to detect and quantify multiple target sequences in a short time. To our knowledge, this technique has not yet been applied to detect plant pathogens.

Portable real-time fluorometers for pathogen detection in the field with limited infrastructure have been developed and applied to different crop systems. In wheat, a LAMP-based foldable microdevice is a promising alternative for detection of pathogens, and its performance was evaluated for detection of *P. oryzae* in rice seeds (Prasannakumar et al., 2021). This approach can be combined with the toothpick DNA extraction method, which saves time and cost for DNA extractions (Wilisiani et al., 2019).

The convenience of LAMP for detection of plant pathogens in wheat should be compared with other isothermal amplification methods. For instance, RPA does not require an initial heating step for DNA denaturation (Baldi and La Porta, 2020). One of the main advantages of RPA is the lower temperature required to conduct the reaction, which is an improvement for field applications where access to electricity may be limited (Panno et al., 2020). Additionally, the use of only two primers in RPA reactions compared to six for LAMP simplifies primer design, and use of the recombinase polymerase lowers detection time. However, the lower reaction temperatures (between 30°C and 55°C) make RPA more prone to non-specific primer binding compared to other isothermal amplification techniques, which can cause amplification of non-target templates (Oliveira et al., 2021). Other limitations of RPA include amplification of only small DNA fragments of less than 1,500 bp with long primers (30–50 nt), which can yield non-specific amplification and a highly variable sensitivity (Ivanov et al., 2021).

RPA has also shown promise for field detection of wheat pathogens. RPA was applied recently for detection of *B. sorokiniana* using the calmodulin gene as a target. The technique showed high specificity when tested against 20 wheat-pathogenic fungal strains. The sensitivity was high with a lower detection limit of 10 pg for pure fungal DNA. RPA was able to detect *B. sorokiniana* directly from field wheat samples (Zhao et al., 2021). Compared to RPA, numerous studies that have successfully conducted LAMP-based approaches to detect pathogen species that affect wheat and other crops are available (Liu et al., 2014; Yasuhara-Bell et al., 2018; Zhang et al., 2018), which support the standardization of LAMP to detect other wheat pathogens. The first multiplex RPA assay coupled with a lateral flow device was recently reported for plant–pathogen detection of bacteria in the genus *Clavibacter* (Larrea-Sarmiento et al., 2021), which opens new possibilities for standardization of this isothermal technique for detection of wheat pathogens.

Rolling-circle amplification (RCA) provides a sensitive method suitable for detection of plant pathogen species. It has been successfully implemented to detect species in the *Fusarium graminearum* complex (Davari et al., 2012). Because RCA does not require expensive instrumentation, it can be suitable for local, point-of-care measurements. A major advantage of RCA over LAMP is the avoidance of carry-over contamination because there is no new 3′-end single-stranded DNA product generated throughout the RCA process (Lau and Botella, 2017). Helicase-dependent amplification (HDA) does not require an initial heat denaturation step and uses uvrD helicase and a reparation protein to activate uvrD. The main disadvantage compared to LAMP, is that HDA demands complex optimization to ensure a coordinated enzyme activity between the helicase and DNA polymerase (Lau and Botella, 2017).

New techniques for detection of plant pathogens in wheat and other crops are currently emerging. The clustered regularly interspaced short palindromic repeats (CRISPR) are an immune system from bacteria and archaea that has been adapted for gene editing in recent years (Wang et al., 2020). CRISPR-related Cas proteins (Cas12 and Cas13) that can recognize and cleave targets complementary to guide sequences (Chen et al., 2018; Gootenberg et al., 2018) give new possibilities for portable and rapid detection. Cas12 and Cas13 proteins have collateral cleavage activities that can detect nucleic acids and return an amplified signal by activating nuclease activity. This technology can be integrated with PCR or LAMP to produce elevated analytical sensitivity for detection (Wang et al., 2020).

A technique for nucleic acid detection named the Specific High-Sensitivity Enzymatic Reporter UnLOCKing (SHERLOCK) system that uses a Cas13a-based molecular detection platform was developed to detect the target sequence by isothermal amplification with RPA/Reverse Transcriptase (RT)-RPA or Loop-mediated Isothermal Amplification (LAMP)/RT-LAMP (Gootenberg et al., 2017). The CRISPR-Cas system for detection involves pre-amplification of the target molecule by isothermal amplification methods, such as LAMP, or RT-LAMP depending on the type of target pathogen genome. Then, the target amplicons are subjected to *in vitro* transcription, followed by the detection of RNA or DNA molecules by a Cas-guided reporter system. A fluorometer or lateral flow device can be used to detect the final products (Gootenberg et al., 2018).

Cas12a ssDNase activation was combined with isothermal amplification to create a method termed DNA endonuclease-targeted CRISPR trans reporter (DETECTR), which achieved attomolar (10^−18^ molecules/ml) sensitivity for DNA detection (Chen et al., 2018). This technique was the foundation for a recently applied method for detection of the *P. oryzae* Triticum lineage using genome-specific primers and Cas12a-mediated technology. Two target markers (*MoT-6098* and *MoT-6099*) were used and its efficiency for detection was evaluated using the LAMP technique. Cas12a along with RPA and nucleic acid lateral flow immunoassay (NALFIA) detected MoT-specific DNA sequences in infected wheat plants with accurate, sensitive, and cost-effective results (Kang et al., 2020). CRISPR-Cas technology is an emerging alternative for rapid diagnosis of wheat diseases that can be integrated with LAMP for application in the field (Kang et al., 2020).

LAMP provides many advantages over other detection methods, but much still needs to be done for detection of diseases in wheat. One need is for LAMP protocols for the many leaf blotch diseases that can co-occur and are difficult to diagnose. Coupled with this would be quantitative and multiplex LAMP that could allow plant pathologists to identify not only the correct species causing disease but also to estimate their relative abundances. Combining the advantages of CRISPR with LAMP approaches for detection of wheat pathogens is another high priority. With the rapid developments of the past few years and availability of these approaches individually the path for future advances is promising.

## Author Contributions

SG-G and SG wrote the manuscript. SG provided significant comments on the article and language editing support. Both authors contributed to the article and approved the submitted version.

## Funding

This work was supported by USDA-ARS research project 5020-21220-019-00D.

## Conflict of Interest

The authors declare that the research was conducted in the absence of any commercial or financial relationships that could be construed as a potential conflict of interest.

## Publisher’s Note

All claims expressed in this article are solely those of the authors and do not necessarily represent those of their affiliated organizations, or those of the publisher, the editors and the reviewers. Any product that may be evaluated in this article, or claim that may be made by its manufacturer, is not guaranteed or endorsed by the publisher.
